# Dynamic Compression Response and Optimization of Stretching–Bending Synergistic Lattices at High Strain Rates

**DOI:** 10.3390/ma19050859

**Published:** 2026-02-25

**Authors:** Xuejiao Gao, Lianchun Long

**Affiliations:** School of Mathematics, Statistics and Mechanics, Beijing University of Technology, Beijing 100124, China; gaoxuejiao@emails.bjut.edu.cn

**Keywords:** lattice structure, strain rate effect, energy absorption, optimized design

## Abstract

Lattice structures are the ideal choice for lightweight, high-strength, and energy-absorbing applications. In this study, the mechanical response of Stretching–Bending Synergistic Lattices (SBSLs) fabricated from 316L stainless steel is investigated under dynamic compression at high strain rates using finite element modeling (FEM), which has been experimentally validated. The results show that the strain rate has a significant influence on specific strength and specific energy absorption (SEA). When the strain rate increases from 100 s^−1^ to 1000 s^−1^, the specific strength increases by 75.6%. A smaller cell height enhances overall impact resistance. The increase in the diameter of the backbone cell rod can simultaneously enhance the SEA and specific strength. To maximize SEA, optimization models for uniform SBSLs and gradient SBSLs are respectively constructed. When the relative density varies, the SEA of the optimized uniform SBSLs has increased by 275.4% and 368.8% compared with the initial SBSL and uniform lattice (UL) designs, respectively. Similarly, the SEA of the gradient SBSLs is enhanced by 154% and 217% compared to the initial design of SBSLs and ULs, respectively. This work deepens understanding of rate-dependent deformation in multi-layer lattices, guiding their design for dynamic loading.

## 1. Introduction

With the increasing demand for lightweight structures in aerospace, defense, and transportation, lattice structures have attracted considerable attention. As ultra-lightweight materials with porous topological configurations, they offer high specific strength, stiffness, and energy absorption capacity. Particularly in applications such as heat exchangers of the spacecraft [1,2], satellite systems [3], orthopedic implants [4], and bionic energy-absorbing structures [5,6], lattice structures demonstrate the design freedom and the potential for functional integration that traditional solid materials cannot achieve. However, engineering structures often encounter complex dynamic loading conditions, such as landing impacts of aircraft, ballistic collisions, and explosive loads, which are characterized by high strain rates and multi-directional forces, posing severe challenges to the impact resistance of lattice structures.

Among various lattice configurations, the stretching-dominated structures typically exhibit higher stiffness and strength [7] (octet truss, face-centered cubic (FCC)), but they are prone to brittle fracture during deformation, while the bending-dominated structures absorb energy by the plastic buckling of the rods and often come with lower plateau stress (body-centered cubic (BCC), bent rod lattice (BDPL)) [8,9]. The Stretching–Bending Synergistic Lattices combine the bending-dominated and stretching-dominated cells ingeniously, aiming to achieve the synergy of high strength and high energy absorption efficiency [10], and have become the current research frontier. A large amount of research on the mechanical behavior of lattice structures under dynamic loads has been conducted. Mueller et al. [11] conducted research on the energy absorption characteristics of random and periodic lattice structures under quasi-static and dynamic loading and tested the influence of the strain rate from 1 to 10^4^ s^−1^ on mechanical properties. The study found that the energy absorption characteristics under quasi-static strain rate loading were usually applicable to low strain rate and medium strain rate conditions. However, under higher strain rate conditions, the performance of the lattice structure will significantly decline, and its performance in different loading directions will also change. Iacolino et al. [12] studied the behavior of two different diameter polylactic acid-based BCC lattice plates under low-speed impact and evaluated their energy absorption by experiment and finite element simulation. The results showed that finer ribs helped to enhance energy dissipation under dynamic loading. Umanzor et al. [13] combined the inherent advantages of additive manufacturing and metal casting to fabricate lattice structures using aluminum A356 and F356 alloys. The performance of the structure was evaluated and simulated, and a path for optimizing the octahedral truss structure was proposed under low strain rate uniaxial compression loading. The results showed that the performance of the optimized A356 alloy structure was approximately 30% higher than the standard design. Uddin et al. [14] fabricated a new lattice structure with a pillar unit in the form of a beam-like configuration using additive manufacturing technology. By low-speed impact (1.54 m/s) experiments, the stiffness, strength, and energy absorption characteristics of the structure were systematically evaluated, and the failure mechanism and destruction process of the structure were revealed by numerical simulation. The results showed that when the quality was the same, the laminated pyramid lattice structure with beam-like pillars in the stack had better rigidity, durability, and energy absorption capacity than the traditional square rod pillar structure. Under low-speed impact conditions, its strength and energy absorption increased by 34% and 74% respectively. Rodrigo et al. [15] experimentally studied the quasi-static and dynamic compression mechanical behavior of stainless steel (SS 316L) functional gradient lattice structures fabricated by electron beam melting (EBM) technology. The functional gradient lattice structure adopted a body-centered cubic (BCC) configuration, including unidirectional and bidirectional density gradients, and its performance was compared with that uniform lattice structure of the same relative density. The experimental results showed that under the selected loading conditions, the new bidirectional density gradient lattice had the highest plateau stress and energy absorption capacity compared to the uniform lattice and unidirectional density gradient lattice. Tian et al. [16] proposed a new lattice structure design strategy by the multi-jet fusion (MJF) additive manufacturing process, with polyamide 12 (PA12) powder as the raw material, and fabricated a programmable heterogeneous layered lattice structure composed of alternating topological arrangements of face-centered cubic (FCC) and body-centered cubic (BCC) lattice sheets, achieving dual mechanical protection. Ye et al. [17] developed a process for transforming fully dense, three-dimensional printed polymer beams into graphite-like carbon hollow tube-in-tube morphology. By the compression experiment and numerical simulation, the findings indicated that as the density decreases, the changes in the tubular structure significantly slowed down the reduction in stiffness, achieving an ideal material structure with high modulus and low density, as well as high modulus and high damping.

Although significant research has focused on the quasi-static behavior of lattice structures, the influence mechanism under dynamic compression remains largely unexplored at high strain rates. Understanding this behavior is critical for the development of next-generation protective structures where energy absorption under impact loading is paramount. This work fills this gap by introducing a high-fidelity FEM, validated experimentally, to elucidate the strain-rate sensitivity of lattice structures. In our previous work [18], we studied the dynamic compression response of the Stretching–Bending Synergistic Lattices (SBSLs) and analyzed the deformation mode and energy absorption mechanism. It was found that SBSLs has better energy absorption characteristics than uniform lattices (ULs) under dynamic compression loading. In this study, the dynamic compression behavior of SBSLs was studied at high strain rates by numerical simulation, obtaining the stress–strain responses and revealing the strain rate strengthening mechanism. Furthermore, the effect of the cell geometric parameters of SBSLs on the structural impact resistance performance was analyzed. To enhance the SEA, SBSL configuration was systematically optimized.

## 2. Materials and Methods

### 2.1. System Geometry

**Figure 1:**
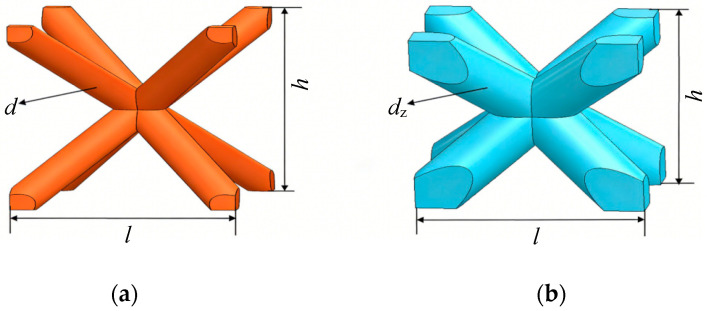
Structure diagram of lattice cells: (**a**) matrix cells of SBSLs, (**b**) backbone cells of SBSLs.

**Figure 2:**
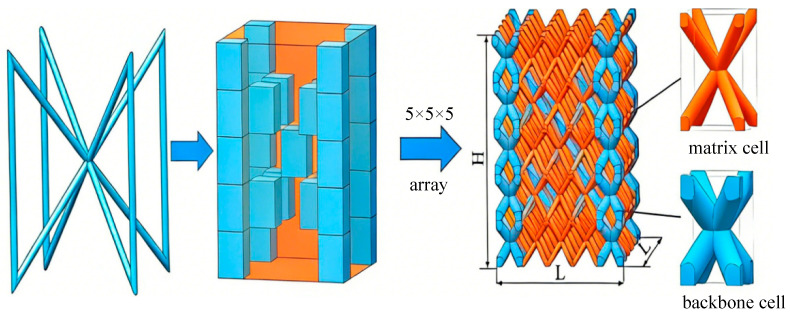
Architecture design of the SBSLs [19].

In our previous work, Stretching–Bending Synergistic Lattices (SBSLs) have been proven to show better energy absorption capacity than well-known uniform lattices (ULs) [18]. SBSLs comprise two distinct types of unit cells: matrix cells and backbone cells. As illustrated in Figure 1a,b, both cells adopt a Body-Centered Cubic (BCC) configuration, differing in the diameter of their constituent struts, while all four struts within a single cell are the same diameter. Each unit cell is defined by three independent geometric parameters: the horizontal side length (*l*), the strut diameter (*d*), and the cell height (*h*). The horizontal side length is equal to the vertical side length, and all struts within a cell have identical diameters. Two cell types are arranged as shown in Figure 2 to form SBSLs. This structure is fabricated in a 5 × 5 × 5 periodic array (5 cells along each of the X, Y, and Z directions), resulting in a total of 125 cells, which constitute a core layer with dimensions of 25 × 25 × 25$\sqrt{2}$ mm (length, width and height). The specific cell geometric parameters are listed in Table 1. The relationship between the rod diameters of the cells and the relative density ($\bar{\rho }$) of SBSLs is established by fitted curves [18]. For the subsequent comparative analysis of impact resistance performance, uniform lattices (ULs) are also constructed. These ULs maintain the same overall shape, dimensions, and relative density, but consist solely of cells with the same cell rod diameter.

### 2.2. Finite Element Modeling

**Figure 3:**
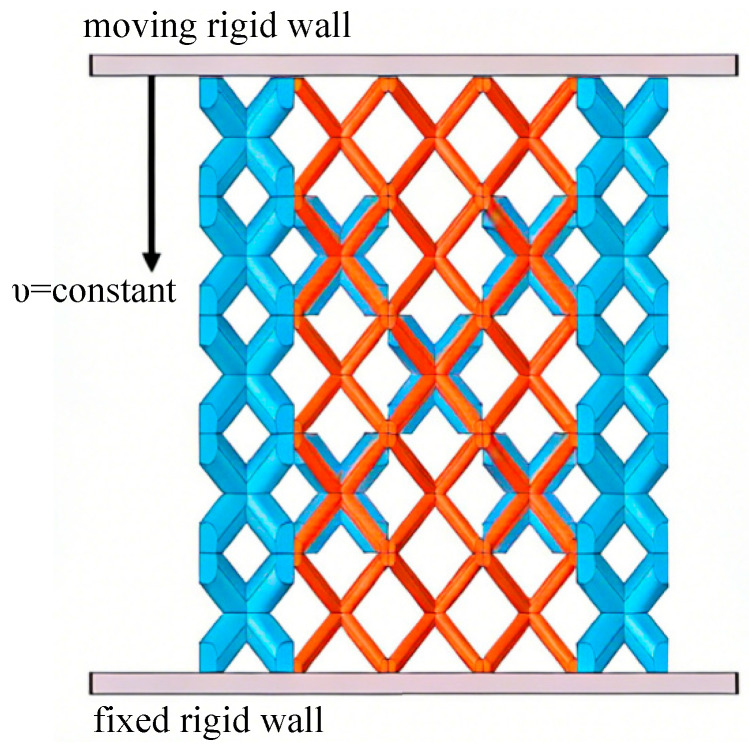
Dynamic compression finite element model of SBSLs [19].

In this work, all finite element simulations (FEM) are performed using the nonlinear explicit solver to simulate the dynamic compression responses. In our previous studies, this simulation method has been compared and verified with experiments, and the results of both are basically consistent [18]. The simulation model is depicted as Figure 3. A rigid plate moving uniformly downward is connected at the top of SBSLs, while the lower end is a fixed rigid plate.

The FEM settings are consistent with those used in our previous studies [18,19]. The general contact algorithm is employed, with a tangential friction coefficient of 0.3 and a normal contact of “hard” type. The mesh is designed to include three layers of hexahedral (C3D8) elements in the radial direction. To prevent excessive distortion of the lattice beam during deformation, the unit cells are meshed using first-order hexahedral solid elements (C3D8) with distortion control set to a length ratio of 0.1. The stability check of time increment is carried out. A convergence analysis of the SBSLs model in Table 1 is conducted for the grid cells. The mesh sizes of the matrix cell are set at 0.16, 0.26, and 0.36, respectively. The influence of grid size on the results is studied, and the error is less than 1.5%. Ultimately a grid of 0.26 is adopted, and the total number of meshes is 295,766. The C3D8 does not require hourglass control because it is a fully integrated unit.

The material is 316L stainless steel, $\rho_{s}$ is the material density, E is the Young’s modulus, and μ is the Poisson’s ratio. The Johnson–Cook constitutive model is adopted as expressed in Equation (1). The specific parameters are shown in Table 2 [20].

(1) $$\bar{\sigma }=\left[A+B{\left(\varepsilon_{p}\right)}^{n}\right]\left[1+C\ln\left(\frac{\dot{\varepsilon }}{{\dot{\varepsilon }}_{0}}\right)\right]\left[1-{\left(\frac{T-T_{r}}{T_{m}-T_{r}}\right)}^{m}\right]$$

Here, the Johnson–Cook (J-C) constitutive model parameters *A*, *B*, *C*, *n*, and *m* are constants determined from Split Hopkinson Pressure Bar experiments. $\overset{-}{\sigma }$ is the flow stress, $\varepsilon_{p}$ is the equivalent strain, $\dot{\varepsilon }$ is the plastic strain rate, ${\dot{\varepsilon }}_{0}$ is the reference plastic strain rate (${\dot{\varepsilon }}_{0}$ = 1/s), *T* is the actual temperature of the testing sample, *T*_r_ is the room temperature, and *T*_m_ is the melting temperature.

The strain (ε) is the same in all models, and the strain rate ($\dot{\varepsilon }$) can be adjusted by altering the compression time (*t*) in the formula (4). The stress, strain, and strain rate of lattice structures can be obtained by the following formulas:

(2) $$\sigma =\frac{F}{A}=\frac{F}{L\times L}$$

(3) $$\varepsilon =\frac{\Delta H}{H}$$

(4) $$\dot{\varepsilon }=\frac{\varepsilon }{t}$$

where *F* represents the reaction force of the moving rigid plate, *L* and *H* are the length and height of lattice structure (as shown in Figure 3), *t* is the loading time, and ΔH is the displacement of the moving rigid plate.

The impact resistance and crashworthiness are primarily assessed by two key indicators: total energy absorption (*EA*) and peak stress ($\sigma_{p}$). In the context of lightweight design, superior performance is characterized by a high specific energy absorption (*SEA*), which indicates efficient energy absorption per unit mass, and a high specific strength ($\sigma_{sp}$), which reflects the bearing capacity. Consequently, The *SEA* and $\sigma_{sp}$ serve as principal metrics for evaluating the energy absorption effectiveness and structural bearing capacity. The expressions [18] are provided as follows:

(5) $$SEA=\frac{EA}{m}=\frac{\int_{0}^{H_{w}}Fds}{m}$$

(6) $$\sigma_{sp}=\frac{\sigma_{p}}{\rho }=\frac{\sigma_{p}}{\bar{\rho }\rho_{s}}$$

(7) $$\sigma_{p}=\;\max\left\{\sigma_{y},\sigma_{f}\right\}$$

where *EA* is the total energy absorption of the structure, *m* is the total mass of the lattice structure, *H*_w_ = *H*$\varepsilon_{d}$ is the axial displacement corresponding to the densification strain, $\varepsilon_{d}$ is the densification strain, $\sigma_{y}$ is the peak stress in the linear elastic stage, $\sigma_{f}$ is the plateau stress, $\sigma_{p}$ is the peak stress, $\rho_{s}$ is the density of the base material, and ρ is the density of the structure.

## 3. Results and Discussion

### 3.1. Dynamic Mechanical Response at Different Strain Rates

**Figure 4:**
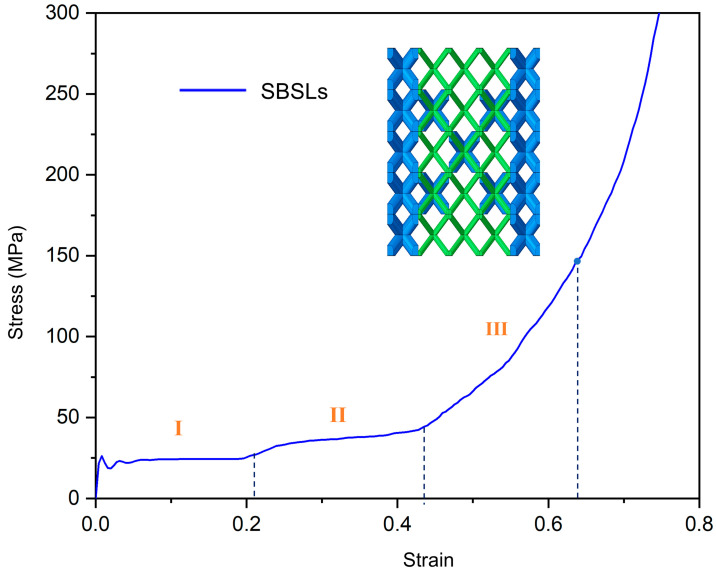
The stress–strain curves of SBSLs at $\dot{\varepsilon }$ = 480 s^−1^.

In our previous work [18,19], the stress–strain curves of SBSLs have similar shapes. The points marked by dots represent densification points, which are determined using the double tangent method [21] as shown in Figure 4. SBSLs exhibit three compression stages: stress plateau (Stage I), plateau raising (Stage II), and stress climbing (Stage III). The peak stress ($\sigma_{p}$) is the first peak point reached during the stress plateau stage (Stage I). The plateau stress ($\sigma_{y}$) is defined as the average stress from the first point after the peak stress to the end point of the stress plateau stage (Stage I).

**Figure 5:**
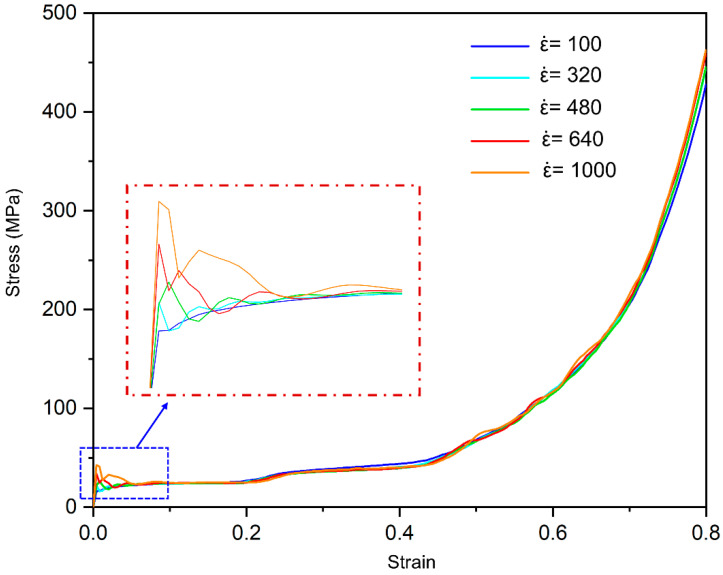
The stress–strain curves of SBSLs at five different strain rates ($\dot{\varepsilon }$ = 100, 320, 480, 640, 1000 s^−1^).

Based on FEM in Section 2.2, the stress–strain curves of SBSLs (with geometric parameters specified in Table 1) at five different strain rates ($\dot{\varepsilon }$ = 100, 320, 480, 640, 1000 s^−1^) under dynamic compression are obtained, as shown in Figure 5. In the initial elastic stage with a small strain (*ε* < 0.004), the stress increases approximately linearly with the strain. The initial peak stress increases significantly with the increase in the strain rate. The peak stress increases from 16.42 MPa at a strain rate of 100 s^−1^ to 41.19 MPa at a strain rate of 1000 s^−1^, corresponding to a 150% rise. As the strain increases, the curve enters a long stress plateau region, which corresponds to the failure modes such as buckling, plastic yielding or fracture of SBSLs, leading to the gradual collapse. The plateau stress also increases with the increase in the strain rate, from 24.43 MPa to 25.85 MPa, which is an increase of 5.8%. It reveals that the peak strengthening is primarily material-controlled near the peak stress period and that the plateau strengthening is structurally controlled after the peak stress fluctuation period. When the $\dot{\varepsilon }$ = 1000, the inertia stabilization effect and material rate sensitivity work together in coupling near the peak stress. When the strain continues to increase, the curve begins to rise steeply, indicating that the structure has been basically compacted and enters densification (*ε* > 0.64), with a sharp increase in stress. As the strain rate changes from low to high (from 100 s^−1^ to 1000 s^−1^), the entire stress–strain curve shows a systematic upward shift in the early stage, with both the peak stress and the plateau stress significantly increasing, indicating that SBSLs have a stronger load-bearing capacity at high strain rates, demonstrating a dynamic strengthening effect. After the curve stabilizes, the influence of the strain rate is relatively small.

**Figure 6:**
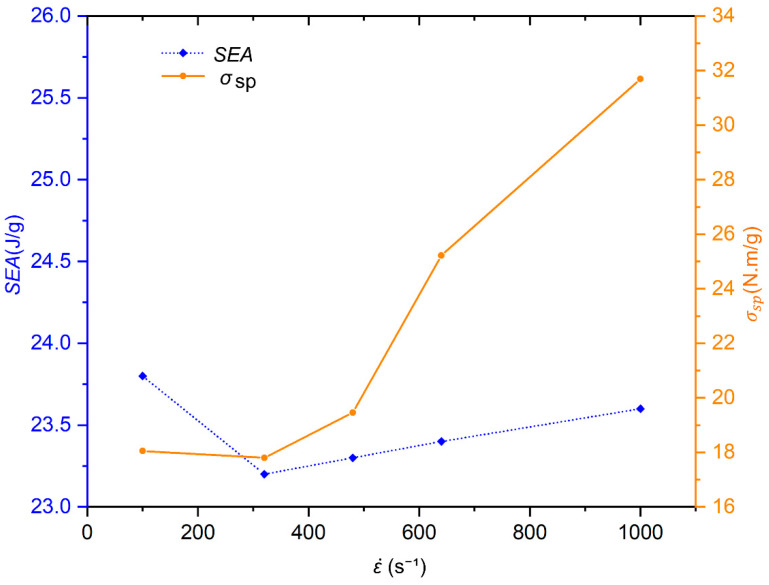
The *SEA* and $\sigma_{sp}$ of the SBSLs at five different strain rates ($\dot{\varepsilon }$ = 100, 320, 480, 640, 1000 s^−1^).

The strain rate has a certain influence on the energy absorption effect and load-bearing capacity of SBSLs. As can be seen from Figure 6, the $\sigma_{sp}$ shows a strong strain rate strengthening effect. With the strain rate increasing from 100 s^−1^ to 1000 s^−1^, the $\sigma_{sp}\;$ sharply rises from 18.05 N·m/g to approximately 31.69 N·m/g, an increase of 75.6%. It demonstrates that the material’s dynamic strength increases with the increase in the strain rate, further verifying that the material has superior load-bearing and anti-impact potential in medium- and high-strain-rate loading. The *SEA* also shows strain rate sensitivity. As the strain rate increases from 100 s^−1^ to 1000 s^−1^, the *SEA* first decreases and then increases. Particularly in the medium- and high-strain-rate range (480~1000 s^−1^), the *SEA* shows a nearly linear growth trend, indicating that the material has a more excellent energy absorption capacity at high strain rates in this range.

**Figure 7:**
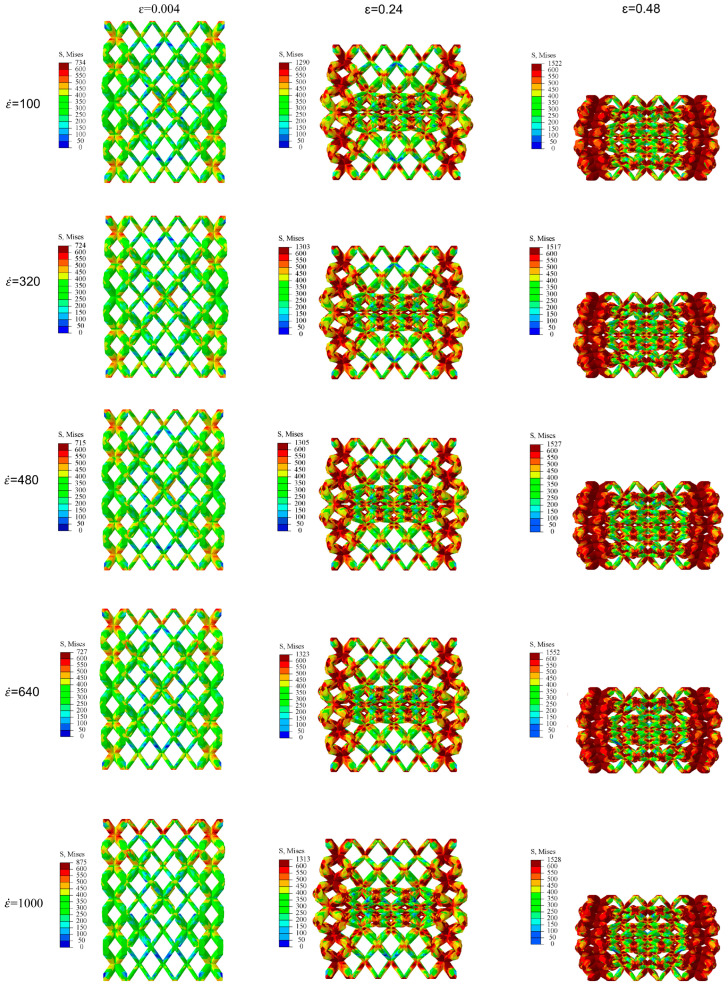
The stress cloud diagram of SBSLs at five different strain rates ($\dot{\varepsilon }$ = 100, 320, 480, 640, 1000 s^−1^).

The overall deformation process and stress distribution of SBSLs under dynamic compression at different strain rates are shown in Figure 7. During the small strain stage (ε = 0.004), the stress distribution is generally an “evenly low-stress field”, with only local geometric discontinuities (such as the edges of the components and interfaces) showing significant stress. The peak Mises stress of the structure under high strain rate (1000 s^−1^) significantly increases, indicating that a strong strain rate strengthening effect occurs in the initial stage of structural deformation. As the deformation of the structure increases (ε = 0.24), the stress concentration area begins to appear. The phenomenon of “more significant stress concentration” under high strain rate occurs, the lag time of dislocation motion under high strain rate is accelerated, and the stress level under the same strain becomes higher. As the deformation gradually increases, the stress concentration area expands further, and the stress peaks of different strain rate cloud diagrams converge.

### 3.2. Effect of Cell Parameters

The geometric parameters of SBSLs are important parameters that affect the impact resistance performance. Based on the finite element simulation analysis in Section 2.2, various SBSLs simulation models are established. When the cell length, width (*l* = 5 mm) and strain rate ($\dot{\varepsilon }$ = 1000) remain unchanged. The effect of three parameters, namely cell height *h*, matrix cells rod diameter *d*, and backbone cells rod diameter *d*_z_, on *SEA* and $\sigma_{\mathrm{sp}}$ are studied respectively.

**Figure 8:**
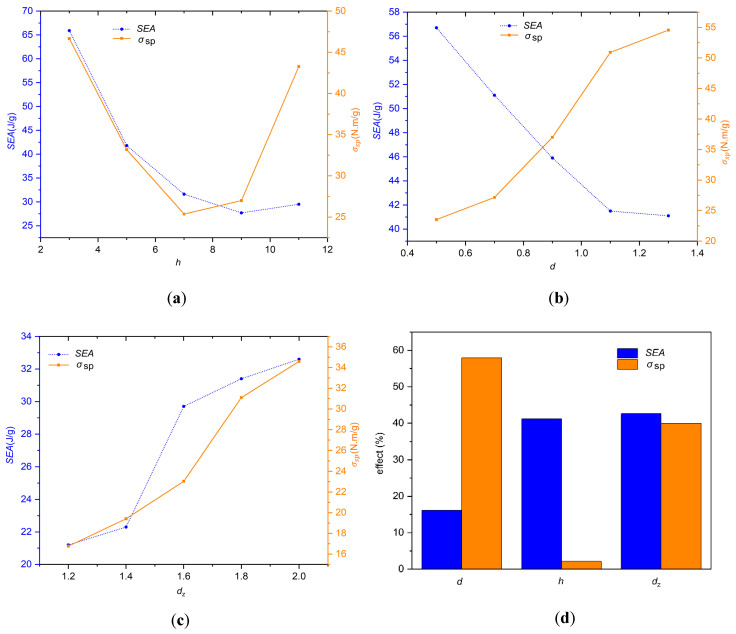
The influence of cell geometric parameters on impact resistance performance: (**a**) The *SEA* and $\sigma_{\mathrm{sp}}$ with different *h*, (**b**) the *SEA* and $\sigma_{\mathrm{sp}}$ with different *d*, (**c**) the *SEA* and $\sigma_{\mathrm{sp}}$ with different *d_z_*, and (**d**) the contribution ratios of *h*, *d*, *d_z_* to the *SEA* and $\sigma_{\mathrm{sp}}$.

The *SEA* and $\sigma_{\mathrm{sp}}$ of SBSLs with different cell heights *h* (*h* = 3, 5, 7, 9, and 11 mm) are shown in Figure 8a. As *h* increases, the *SEA* and $\sigma_{\mathrm{sp}}$ both show a trend of first decreasing and then increasing. When *h* = 3 mm, both the *SEA* and $\sigma_{\mathrm{sp}}$ are at their maximum values. It confirms that in this range, a lower cell height is beneficial for improving the structural impact resistance. The *SEA* for different diameters *d* (*d* = 0.5, 0.7, 0.9, 1.1, and 1.3 mm) of matrix cells rod are shown in Figure 8b. As *d* increases, the *SEA* gradually decreases, while the $\sigma_{\mathrm{sp}}\;$ gradually increases. It shows that a larger *d* is beneficial for load-bearing capacity, while a smaller *d* is beneficial for energy absorption. The *SEA* for different diameters *d*_z_ (*d*_z_ = 1.2, 1.4, 1.6, 1.8, and 2 mm) of backbone cells rod are shown in Figure 8c. As *d*_z_ increases, the *SEA* and $\sigma_{\mathrm{sp}}$ both show a gradually increasing trend. As *d*_z_ increases, both *SEA* and $\sigma_{\mathrm{sp}}$ increase steadily. The contribution ratios of three parameters (*h*, *d*, and *d*_z_) to the *SEA* are shown in Figure 8d. It can be observed that all three parameters have a significant impact on the *SEA*, while the impact of cell height *h* on the $\sigma_{\mathrm{sp}}$ is relatively small.

## 4. Optimizations

Based on the effect of cell parameters in Section 3.2, under the dynamic compression at a high strain rate (1000 s^−1^), the three parameters (*h, d, d*_z_) have a significant impact on the *SEA* of the SBSLs. To obtain the SBSLs with the optimal *SEA*, the optimization models for uniform SBSLs and gradient SBSLs with the objective of maximizing the *SEA* are established.

This study adopts the Design of Experiments (DOE) approach combined with a surrogate modeling technique. Sampling points are generated using the Optimal Latin Hypercube method, comprising 45 points for uniform SBSLs and 60 points for gradient SBSLs. Finite element models corresponding to these sampling points are constructed and simulated in Abaqus to compute their *SEA*. Due to the highly nonlinear behavior of structures under dynamic compressive loading, performing full numerical simulations for all candidate designs would be computationally expensive. To perform the optimization, the radial basis function (RBF) surrogate model is employed in conjunction with the sequential quadratic programming (NLPQLP) algorithm.

### 4.1. Optimization of Uniform SBSLs

Keep the diameter of the same type of cell rods the same for any layer, uniform SBSLs are optimized. With the maximization of the *SEA* as the optimization objective, the design variables are the cell height *h*, the diameter *d* of the matrix cells rod, and the diameter *d*_z_ of the backbone cells rod. The optimization model parameters are shown in Table 3. The optimization model is established as follows:

$$\begin{array}{ll} \mathrm{Find}: & \\ & \;\;x={\left[h,d,d_{z}\right]}^{T}\\ \mathrm{To}\mathrm{optimize}: & \\ & maxF(x)=Maximize\;SEA(h,d,d_{z})\\ \mathrm{Subject}\mathrm{to}: & \\ & \;\;g_{1}\left(x\right):h^{min}\leq h\leq h^{max}\\ & \;\;g_{2}\left(x\right):d^{min}\leq d\leq d^{max} \end{array}$$

(8) $$\begin{array}{cc} & \;\;g_{3}\left(x\right):d_{z}^{min}\leq d_{z}\leq d_{z}^{max} \end{array}$$

**Figure 9:**
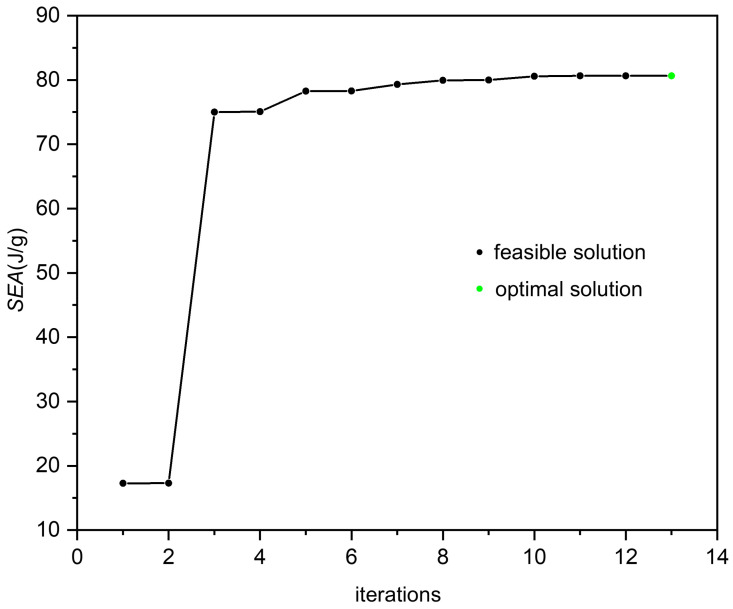
The iterative process of optimization for uniform SBSLs.

An RBF surrogate model is constructed based on 45 sample points, and the fitting relationship between the *h*, *d*, *d*_z_ and the *SEA* is established. To verify the accuracy of the approximate model, 10 points are randomly selected from the samples. The fitting degree evaluation coefficient *R*^2^ [22] and the Root Mean Square Error (RMSE) [23] are used, which are 0.926 and 0.11382, respectively. Given the high accuracy of the approximate model, it can reliably substitute the numerical simulation process for optimization. Consequently, the NLPQLP algorithm is employed to obtain the structure with the optimal *SEA*. Figure 9 shows the optimization process. The green point represents the optimal solution, and the black points represent the feasible solutions. The optimization curves converge and stabilize, indicating robust convergence behavior. The green point represents the final optimization result, and the specific parameters are shown in Table 4.

Reconstruct the finite element model based on the optimized parameters and conduct the analysis. Compare and verify the *SEA* of optimization result with that of the initial model. The final analysis results are shown in Table 4. When the relative density varies, compared with the initial design of uniform SBSLs and ULs, whose parameters are shown in Table 1, the *SEA* of the optimized uniform SBSLs has increased by 275.4% and 368.8%, respectively.

### 4.2. Optimization of Gradient SBSLs

The gradient design of the lattice structure can dissipate energy layer by layer in impact protection, thereby improving the energy absorption efficiency. In this section, the gradient design is carried out for SBSLs where the diameters of two types cells rod in each layer are independently variable. From the stress cloud diagram in Figure 7, it can be observed that under dynamic compression, the deformation of the five-layer of SBSLs is symmetrically distributed around the middle layer. Therefore, the gradient optimization is centered on the middle layer, and the diameters of two types of cell rods in the symmetrical layers remain consistent. The optimization objective is to maximize the *SEA* of SBSLs. The design variables are the diameters *d*_ij_ of two types cells rods, where *i* represents the layer number and *j* represents the cell type. The digit 1 represents matrix cells and 2 represents backbone cells. The optimization model parameters are shown in Table 5. The optimization model is established as follows:

$$\begin{array}{ll} \mathrm{Find}: & \\ & \;\;\;\;x={\left[d_{ij}\right]}^{T}\\ \mathrm{To}\mathrm{Optimize}: & \\ & \;\;\;\;\max F\left(x\right)=Maximize\;SEA(d_{ij})\\ \mathrm{Subject}\mathrm{to}: & \\ & \;\;\;\;g_{1}\left(x\right):{d_{i1}}^{min}\leq d_{i1}\leq {d_{i1}}^{max} \end{array}$$

(9) $$\begin{array}{cc} & \;\;\;\;g_{2}\left(x\right):d_{i2}^{min}\leq d_{i2}\leq d_{i2}^{max} \end{array}$$

**Figure 10:**
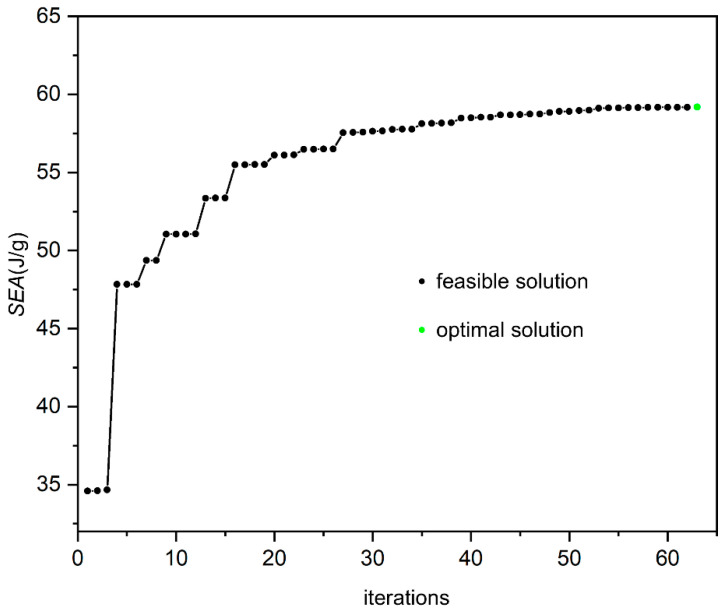
The iterative process of optimization for gradient SBSLs.

Based on 60 sample points, an RBF surrogate model is constructed. By randomly selecting 15 points from sample points, the fitting degree evaluation coefficient *R*^2^ and the Root Mean Square Error (RMSE) are 0.911 and 0.11782, respectively. The accuracy of the approximate model is high and can be used to replace the numerical simulation process for optimization. Based on the NLPQLP algorithm, gradient BSBSLs with the optimal *SEA* are obtained. Figure 10 illustrates the optimization process. The black points represent the feasible solutions, and the green points are the final optimization results. The optimization curve converges and stabilizes. The model is reconstructed according to the optimized parameters and simulation verification is carried out. The specific values are shown in Table 6. The simulation results show that for gradient SBSLs, when the relative density varies, the *SEA* of the optimized gradient SBSLs improves by 154% and 217% compared to the initial design of SBSLs and ULs.

## 5. Conclusions

This work proposes the lattice structure named Stretching–Bending Synergistic Lattices (SBSLs), which comprises two cell types: matrix cells and backbone cells. The dynamic response of SBSLs under dynamic compression at different strain rates and the impact resistance performance were studied by the FEM. This study investigated three main aspects. First, it examined the deformation mode and energy absorption mechanism of SBSLs under dynamic compression at various strain rates. Second, it analyzed how the cell height and two types of cell diameters influenced the dynamic response and energy absorption characteristics. Third, with the goal of maximizing the *SEA*, the optimization models for uniform SBSLs and gradient BSBSLs were established and optimized. This study advances the fundamental understanding of the rate-dependent mechanical behavior of the SBSLs under dynamic compression, elucidating their distinct deformation and energy dissipation mechanisms across varying strain rates. By establishing a comprehensive analysis and optimization framework for impact resistance, this work provides novel design strategies for achieving superior load-bearing and energy absorption in lightweight architectures. The findings offer practical pathways for the deployment of high-performance SBSL structures in critical applications such as spacecraft protection systems and load-bearing components in unmanned equipment, thereby enhancing the structural reliability and impact resistance of lightweight lattice materials under extreme loading conditions. The main conclusions are as follows:

(1) The dynamic response of SBSLs under dynamic compression at different strain rates was studied. It was found that the strain rate has a certain influence on the energy absorption effect and load-bearing capacity. The specific strength and *SEA* both exhibit a strong strain-rate strengthening effect. As the strain rate increases from 100 s^−1^ to 1000 s^−1^, the specific strength continuously rises, with an increase of 75.6%. The *SEA* first decreases and then increases, especially in the medium- to high-strain-rate range (480~1000 s^−1^), showing a nearly linear growth trend.
(2) The effect of cell geometric parameters of SBSLs was analyzed. As the cell height *h* increased, the *SEA* and specific strength both showed a trend of first decreasing and then increasing. When *h* = 3 mm, the *SEA* and specific strength both reached their maximum values. This indicates that a lower cell height *h* is beneficial for improving the structural impact resistance. As the diameter *d* of the matrix cells rods increases, the *SEA* gradually decreases, while the specific strength gradually increases. This shows that a larger *d* is beneficial for the load-bearing capacity, while a smaller *d* is beneficial for energy absorption. As the diameter *d*_z_ of backbone cell rods increases, the energy absorption effect and load-bearing capacity both show an enhancing trend. Therefore, increasing *d*_z_ is beneficial for improving the structural impact resistance.
(3) Two optimization models for uniform SBSLs and gradient BSBSLs were established, one with two cell rod diameters (*d* and *d*_z_) and cell height *h* as optimization variables and the other with two cell rod diameters (*d* and *d*_z_) as optimization variables, and take maximizing the *SEA* as the optimization objective. The optimal Latin Hypercube experimental design method and RBF surrogate model method were adopted, and the NLPQLP algorithm was used for optimization. For uniform SBSLs, compared with the initial design of uniform SBSLs and ULs, the *SEA* of the optimized structure has increased by 275.4% and 368.8%, respectively. For gradient SBSLs, with a constant height, the *SEA* of the optimized structure improves by 154% and 217% compared to the initial design of SBSLs and ULs, respectively.

## Figures and Tables

**Table 1:** Geometry parameters for matrix cell and backbone cell.

Cell Type	Rod Diameter [mm]	Cell Size [mm]	Relative Density [%]	Volume [mm^3^]	Quantity
matrix cell of SBSLs	0.805	$5\;\times \;5\;\times \;5\sqrt{2}$	10	17.102	96
backbone cell of SBSLs	1.783	$5\;\times \;5\;\times \;5\sqrt{2}$	40	68.698	29
cell of ULs	1.0765	$5\;\times \;5\;\times \;5\sqrt{2}$	16.96	29.523	125

**Table 2:** J-C model parameters and material properties for 316L stainless steel [20].

Parameters	*A* [MPa]	*B* [MPa]	*C*	*n*	*m*	*T*_m_ [K]	*T*_r_ [K]	$\rho_{s}$ [g/cm^3^]	*E* [GPa]	μ
value	305	1161	0.01	0.61	0.517	1672	293	7.98	210	0.3

**Table 3:** The parameters of the optimization model for uniform SBSLs.

Parameters	$\dot{\varepsilon }$ [s^−1^]	*l* [mm]	*h* [mm]	d [mm]	$d_{z}$ [mm]
value	1000	5	3~11	0.5~1.2	1.3~2

**Table 4:** Optimization results of uniform SBSLS.

Group	Matrix Cells Rod Diameter [mm]	Backbone Cells Rod Diameter [mm]	Cell Height [mm]	Relative Density [%]	*SEA* [J/g]
initial model	0.805	1.783	5$\sqrt{2}$	16.96	23.6
	1.0765	1.0765	5$\sqrt{2}$	16.96	18.9
optimal configuration	1.158	1.429	3		80.64
simulation results				22.17	88.6

**Table 5:** The parameters of the optimization model for gradient SBSLs.

Parameter	$\dot{\varepsilon }$ [s^−1^]	*l* [mm]	*h* [mm]	$d_{i1}$ [mm]	$d_{i2}$ [mm]
value	1000	5	$5\sqrt{2}$	0.5~1.5	1.6~2.6

**Table 6:** Optimization results of gradient SBSLs.

Model	Group	Matrix Cells Rod Diameter [mm]			Backbone Cells Rod Diameter [mm]			Relative Density [%]	*SEA* [J/g]
		*d*_11_	*d*_21_	*d*_31_	*d*_12_	*d*_22_	*d*_32_		
uniform SBSLs	initial model	0.805	0.805	0.805	1.783	1.783	1.783	16.96	23.6
ULs		1.0765			1.0765			16.96	18.9
gradient SBSLs	optimal configuration	0.5	0.839	0.924	1.786	2.295	2.447	19.74	59.9
	simulation results								59.8

## Data Availability

The original contributions presented in this study are included in the article. Further inquiries can be directed to the corresponding author.
